# Methotrexate associated to lipid core nanoparticles improves cardiac allograft vasculopathy and the inflammatory profile in a rabbit heart graft model

**DOI:** 10.1590/1414-431X20176225

**Published:** 2017-08-17

**Authors:** A.I. Fiorelli, D.D. Lourenço-Filho, E.R. Tavares, P.O. Carvalho, A.F. Marques, P.S. Gutierrez, R.C. Maranhão, N.A.G. Stolf

**Affiliations:** 1 Instituto do Coração, Faculdade de Medicina, Universidade de São Paulo, São Paulo, SP, Brasil; 2 Faculdade de Ciências Farmacêuticas, Universidade de São Paulo, São Paulo, SP, Brasil

**Keywords:** Nanoemulsions, Solid lipid particles, Methotrexate, Inflammatory mediators, LDL receptors

## Abstract

Coronary allograft vasculopathy is an inflammatory-proliferative process that compromises the long-term success of heart transplantation and has no effective treatment. A lipid nanoemulsion (LDE) can carry chemotherapeutic agents in the circulation and concentrates them in the heart graft. The aim of the study was to investigate the effects of methotrexate (MTX) associated to LDE. Rabbits fed a 0.5% cholesterol diet and submitted to heterotopic heart transplantation were treated with cyclosporine A (10 mg·kg^–1^·day^–1^ orally) and allocated to treatment with intravenous LDE-MTX (4 mg/kg, weekly, n=10) or with weekly intravenous saline solution (control group, n=10), beginning on the day of surgery. Animals were euthanized 6 weeks later. Compared to controls, grafts of LDE-MTX treated rabbits showed 20% reduction of coronary stenosis, with a four-fold increase in vessel lumen and 80% reduction of macrophage staining in grafts. Necrosis was attenuated by LDE-MTX. Native hearts of both LDE-MTX and Control groups were apparently normal. Gene expression of lipoprotein receptors was significantly greater in grafts compared to native hearts. In LDE-MTX group, gene expression of the pro-inflammatory factors tumor necrosis factor-α, monocyte chemoattractant protein-1, interleukin-18, vascular cell adhesion molecule-1, and matrix metalloproteinase-12 was strongly diminished whereas expression of anti-inflammatory interleukin-10 increased. LDE-MTX promoted improvement of the cardiac allograft vasculopathy and diminished inflammation in heart grafts.

## Introduction

Heart transplantation is presently the gold standard treatment for patients in end-stage heart failure, to whom this procedure may offer several years of good quality of life (1). Heart transplantation is in fact the only available procedure that has the capability to restore the normal hemodynamic status of those patients. However, the long-term success of heart transplantation may be hampered by a typical coronary allograft vasculopathy, an inflammatory-proliferative process that is actually an accelerated atherosclerosis-like process affecting the coronary arteries of the grafted heart (2).

Several immunological and non-immunological factors have been implicated in the genesis of the disease, such as antibody-mediated rejection, human leukocyte antigen mismatch, cytomegalovirus infection, mode of donor brain death, and also classical cardiovascular risk factors such as dyslipidemia, insulin resistance and hypertension (3).

Cardiac allograft vasculopathy is characterized by diffuse and concentric arterial obstruction rather than the focal aspect of common atherosclerosis. As the arterial obstruction occurs mainly in the distal vessels, percutaneous coronary interventions and coronary artery bypass are ineffective as treatment modalities, so that no therapy for the disease is currently at hand (4). Achievement of an efficacious treatment for this condition is thus of paramount importance for the optimization of the heart transplantation procedure.

In a previous study, we showed that nanoemulsions (LDE) that resemble the lipid structure of low-density lipoprotein (LDL) and bind to LDL receptors (5,6) can be used as drug carriers. LDE has the ability to concentrate in lesioned arteries of rabbits with atherosclerosis induced by cholesterol-rich diet (7). Treatment of the animals with chemotherapeutic agents used in cancer treatment, such as paclitaxel, etoposide and methotrexate (MTX) associated to LDE resulted in marked reduction of the atheromatous lesions (7–9). In the surgical model of rabbits with heterotopic heart graft, LDE concentrated four-fold more in the heart graft than in the native heart. Treatment of the rabbits with paclitaxel carried in LDE markedly reduced coronary stenosis in the grafted heart (10).

As tested in experimental animals (11,12) and also in clinical studies enrolling patients with advanced cancers (13–16), association of antineoplastic drugs to LDE pronouncedly reduces the high toxicity of those drugs, thus circumventing the major drawback to their use in the therapy of cardiovascular diseases.

MTX is an anti-proliferative, immunosuppressive and anti-inflammatory agent used in cancer chemotherapy and other diseases such as rheumatoid arthritis and psoriasis. In patients with heart transplantation, MTX has been used as adjuvant in the treatment of refractory rejection (17). MTX promotes the inhibition of DNA replication by inhibiting dihydrofolate reductase that is involved in the synthesis of thymidylate, purines, methionine, and serine (18). It is important to highlight that association to LDE increases by ninety-fold the uptake of MTX by the cells, conceivably because the lipoprotein receptor-mediated endocytosis pathway is much more efficient than the folate receptor to internalize the drug (19).

Based on our previous finding that the treatment with MTX associated to LDE was remarkably efficient in inhibiting the atherosclerotic lesions in rabbits (9), we hypothesized whether LDE-methotrexate could also be useful to improve the inflammatory proliferative process that occurs in the grafted heart. Thus, the aim of the current study was to evaluate the effect of LDE-MTX on coronary stenosis and inflammatory process using the experimental model of cholesterol-fed rabbits submitted to heterotopic heart transplantation.

## Material and Methods

### Animals and experimental protocols

Twenty-six male New Zealand white rabbits weighing 3.4±0.6 kg and 26 male New Zealand red rabbits weighing 2.7±0.5 kg (means±SD) were housed in individual cages in a temperature-controlled room (20–22°C) and on a 12-h light/dark cycle during the experimental period. White rabbits were the heart graft recipients and red rabbits were the heart graft donors.

Heterotopic heart grafting surgery was performed as described by Alonso et al. (20). Briefly, right anterior cervicotomy with exposure of carotid artery and jugular vein was made in the receptor rabbit. Meanwhile, the donor rabbit was submitted to median thoracotomy and exposure of the heart; at this point, the animal was heparinized and explantation of the heart was made after topic hypothermia with 4°C ringer lactate. Transplantation was made with previous heparinization of the recipient rabbit through anastomosis of the aorta with the carotid artery and anastomosis of the pulmonary artery with the jugular vein. The recipient rabbits were allocated to two experimental groups: 1) LDE-MTX treated group: rabbits with heterotopic heart transplantation at the cervical position treated with 4 mg/kg body weight methotrexate associated with LDE intravenously, once a week beginning on the day of the procedure for 6 weeks of follow-up. Twelve animals initiated the protocol, but 2 animals died in the first 48 h, resulting in a final number of 10 animals. 2) Control group: control rabbits with heterotopic heart transplantation at the cervical position administered 3 mL saline intravenously once a week for 6 weeks. Fourteen animals initiated the protocol, but 4 animals died in the first 48 h, resulting in a final number of 10 animals. Bleeding in the cervical region was the only observed cause of death in both groups.

Previously, we had observed in rabbits that the injection of LDE alone, without association of methotrexate, had no effect on inflammatory and proliferative processes in atherosclerotic lesions (9). For this reason, a second control group consisting of rabbits treated with LDE alone, without the drug, was not included in the study protocol. Free methotrexate was also not included as a study group of animals because of the drug toxicity, which is attenuated by association with LDE (21).

In this period, all animals were fed regular chow with added 0.5% cholesterol and were treated with 10 mg·kg^–1^·day^–1^ cyclosporine A. Graft beating was monitored by daily local palpation. After the experimental period, rabbits were euthanatized by sodium pentobarbital injection for analysis.

The study protocol was approved by the Animal Ethics Committee of the Universidade de São Paulo, São Paulo, Brazil.

### Plasma lipids and blood cell counting

Blood samples were taken before the surgery and on the day before sacrifice from the marginal ear vein after overnight fasting for determination of total cholesterol, HDL cholesterol and triglycerides by commercial kits (Labtest, Brazil), and for blood cell count.

### Preparation of LDE and association with methotrexate

LDE-MTX was prepared from a lipid mixture composed of 100 mg cholesteryl oleate, 200 mg egg phosphatidylcholine (Lipoid, Germany), 10 mg triglycerides, 12 mg cholesterol and 60 mg of MTX (5,19). The aqueous phase (100 mg of polysorbate 80 and 10 mL of Tris-HCl buffer, pH 8.05) was kept at room temperature. The pre-emulsion was obtained adding the hydrophilic phase to the oil phase by ultrasonic radiation until complete dissolution of the drug. Emulsification of the compounds was obtained by high-pressure homogenization using an Emulsiflex C5 homogenizer (Avestin, Canada). After homogenization at constant temperature, the nanoemulsion was centrifuged at 1800 *g* for 15 min at 4°C to separate the emulsified from unbound MTX. The nanoemulsion was sterilized by passage through 0.22 μm pore filter (Millipore, USA) and kept at 4°C until it was used. The association of MTX to LDE was measured by HPLC before the treatment (22).

### Tissue samples

After the experimental period, both native and grafted hearts were excised and washed with saline solution. A 0.1 g fragment of both hearts was immediately stored in RNA*later*® solution for posterior analysis (Ambion Life Technology, USA). The hearts were then fixed on 10% buffered formalin. After fixation, hearts were transversally cut into three portions. The middle portion that contains all three main artery types - small, medium and large arteries - was sectioned in 5 mm-segments and embedded in paraffin.

### Analysis of coronary artery elastic lamina and lumen

Sections were stained with hematoxylin-eosin and with Verhoeff-van Gieson. The area of the transversal section of coronary arteries was estimated by measuring internal elastic lamina and the vessel lumen area of small, medium and large arteries (23). Three sections were analyzed per heart and all arteries identified in each section (8–10 arteries) were measured. Percentage of stenosis was calculated from the difference between lumen area and the internal elastic lamina area. The measurements were performed by a pathologist blind to the study groups.

### Immunohistochemistry of native and heterologous hearts

Heart sections were labeled with anti-RAM 11 antibody (Dako Cytomation, Denmark) to stain macrophages. The stained area was calculated as percentage of each analyzed field. All measurements were performed using a Nikon Eclipse 80i optical microscope with Image Analysis System NIS-Element AR 3.2 (Nikon, Japan).

### Histological evaluation of myocardium

The inflammatory process of the myocardium of grafted hearts was graded from zero to four according to the presence of inflammatory cells. Recent necrosis was graded from zero to two. Presence or absence of steatosis, calcification and hemorrhage were registered. A pathologist blind to the study groups performed the analysis.

### RNA isolation and cDNA synthesis

Total RNA was isolated from tissues of native and grafted hearts of control and LDE-methotrexate groups using TRIzol® Reagent (Invitrogen Biosystems Life Technology, USA) according to the instructions of the manufacturer. In the recipient animals that were not treated with LDE-MTX, the amount of RNA isolated from the cardiac tissue of each animal was not sufficient for analysis, possibly because of greater presence of necrotic or fibrotic tissue. Therefore, we processed tissue pools instead of individual heart samples to obtain a favorable volume of RNA. After RNA quantification in Nanodrop 2000 Spectrophotometer instrument (Thermo Fisher Scientific Inc., USA), one microgram of total RNA of each pool was reverse-transcribed using the High Capacity RNA-to-cDNA Master Mix (Applied Biosystems Life Technology, USA) according to the manufacturer’s specifications. Each cDNA mixture was diluted 30× with Tris-EDTA buffer and stored at –20°C before analysis.

### Quantitative real-time PCR (qRT-PCR) for gene expression analysis

Gene expression levels of tumor necrosis factor-alpha (TNF-α), monocyte chemoattractant protein (MCP-1), vascular cell adhesion protein 1 (VCAM-1), interleukin-1 beta (IL-1β), IL-10, IL-18, matrix metalloproteinase-9 (MMP-9) and −12 (MMP-12), low-density lipoprotein receptor (LDLR), low-density lipoprotein receptor-related protein-1(LRP-1) and cluster of differentiation 36 (CD36) were quantified in heart tissue using the TaqMan detection system. Relative mRNA expression of target genes was normalized to the endogenous glyceraldehyde-3-phosphate dehydrogenase (GAPDH) gene. The equation 2^-ΔΔCt^ was applied to calculate the relative expression of target genes comparing the mean of the pool of grafted hearts of control and LDE-MTX groups *versus* the mean of the pool of native hearts of control group (24).

All PCR reactions contained 1.2 µL of diluted cDNA with 6 µL of TaqMan Universal PCR Master Mix, 0.6 µL of 20x TaqMan Gene Expression Assays (Applied Biosystems) were performed in duplicates using the StepOne Plus Real-Time PCR System (Applied Biosystems) following the cycle conditions: 50°C for 2 min, initial denaturation at 95°C for 10 min and 40 cycles of 95°C for 15 s and 60°C for 1 min.

### Statistical analysis

Differences in weight, serum lipids and hematological profile of control and LDE-MTX groups at baseline and after treatment were assessed using one-way ANOVA with Tukey post-test. For coronary stenosis measurements and immunohistochemical staining, Student's *t*-test was used to compare grafted hearts and native hearts separately. In all analyses, differences with two-tailed P<0.05 were considered statistically significant. Data are reported as means±SD.

## Results

### Body weight, serum lipids and hematological data

Table 1 shows that, in both the LDE-MTX treated and the control groups, there was no change in body weight from baseline to 6 weeks after the beginning of cholesterol feeding and the heart transplantation surgery. In this period, a ten-fold increase in the serum total cholesterol concentration values occurred (P<0.001) in both groups. On the other hand, HDL cholesterol increased in the controls (P<0.001) but not in the rabbits treated with LDE-MTX, whereas the triglyceride values were unchanged in both groups. Blood cell and leukocyte counts, as well as the differential leukocyte count, were unchanged 6 weeks after the surgery in both LDE-MTX treated and control animals (Table 1).

**Table 1.** Body weight, serum lipids and hematological profile of heart grafted controls (n=10) and heart grafted rabbits treated with lipid nanoemulsion-methotrexate (LDE-MTX) (4 mg·kg body weight ^–1^·week^–1^ during 6 weeks, n=10).

	Controls		LDE-MTX	
	Baseline	6th week	Baseline	6th week
Body weight (kg)	3.7±0.6	3.5±0.8	3.9±0.3	3.7±0.2
Serum lipids (mg/dL)				
Total cholesterol	58±33	563±195* #	49±29	628±197* #
HDL cholesterol	9±5	17±3* +	10±3	14±4§
Triglycerides	194±120	212±99	109±76	169±45
Hematological profile				
Red blood cells (10^9^/mL)	5.2±0.7	4.9±2.0	5.5±1.6	5.0±1.3
Leukocytes (10^6^/mL)	6.3±1.8	6.9±2.1	6.0±2.2	6.7±3.0
Lymphocytes (%)	72.0±8.3	75.0±5.8	76.8±11.1	78.1±4.9
Monocytes (%)	8.3±3.6	8.1±4.5	6.7±2.4	8.4±3.5
Neutrophils (%)	19.7±5.5	16.9±5.4	16.5±10.2	13.7±4.8

All animals were treated with cyclosporine A (10 mg·kg^–1^·day^–1^). Data are reported as means±SD.

* P<0.001 *vs* control baseline;

# P<0.001 *vs* LDE-MTX baseline;

§P<0.05 *vs* control baseline;

+ P<0.01 *vs* LDE-MTX baseline. Statistical analysis was done with one-way ANOVA and Tukey’s post-test.

### Coronary artery measurements

As observed 6 weeks after the transplantation procedure, LDE-MTX treatment reduced the percentage of stenosis of the coronary arteries by roughly 20% (P<0.001), compared to control rabbits (Table 2). The vessel lumen area was four-fold larger in the LDE-MTX treated group than in the controls (P<0.01). It is worthwhile to point out that the coronary arteries of the native hearts of both treated and control groups showed no stenosis (Table 2).

**Table 2.** Coronary artery measurements and percent of macrophage invasion obtained by light microscopy morphometry and immunohistochemistry in heart grafted controls (n=10) and in heart grafted rabbits treated with lipid nanoemulsion-methotrexate (LDE-MTX) (4 mg·kg body weight ^–1^·week^–1^ during 6 weeks, n=10).

	Control		LDE-MTX	
	Native heart	Heart graft	Native heart	Heart graft
Morphometry				
Elastic lamina area (mm^2^)	16.4±5.7	148.6±136.4	12.6±2.8*	179.6±166.2
Arterial lumen area (mm^2^)	16.4±5.7	11.4±12.8	12.6±2.8*	40.4±29.8**
% Stenosis	0	90.8±9.5	0	72.6±11.2***
Immunohistochemistry				
% Macrophage	0.04±0.07	27.13±21.07	0.04±0.09	5.97±4.05**

All animals were treated with cyclosporine A (10 mg·kg^–1^·day^–1^). Data are reported as means±SD.

* P<0.05 *vs* control native heart;

**P<0.01 *vs* control heart graft;

***P<0.001 *vs* control heart graft. Statistical analysis was done with Students’ *t*-test.

### Myocardium histology

**Figure 1.**
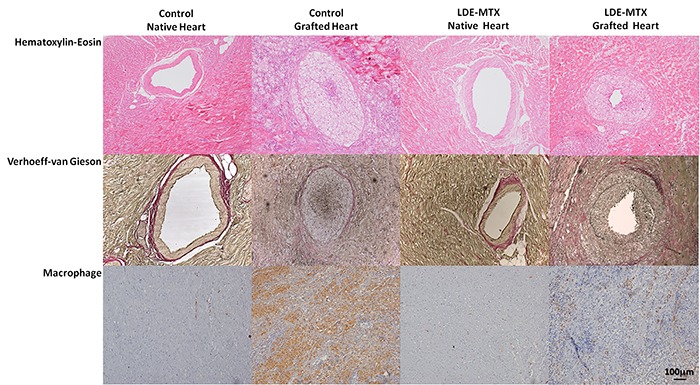
Native and grafted hearts of control rabbits, and native and grafted hearts of lipid nanoemulsion-methotrexate (LDE-MTX) treated rabbits stained with hematoxylin-eosin, Verhoeff-van Gieson and immunostaining for macrophages. Magnification 100×.

In the grafted hearts stained by hematoxylin-eosin, the intensity of inflammation in the myocardium was apparently not different in LDE-MTX treated and control rabbits. However, by immunostaining (Figure 1), LDE-MTX treatment achieved a four-fold reduction of the area stained for macrophages compared to the controls (Table 2).

Necrosis was less intense in the treated animals than in controls. Grade zero necrosis was observed in 4 LDE-MTX treated animals and in only 1 of the control rabbits. Grade 1 necrosis was found in 5 LDE-MTX treated and in 5 controls. Grade 2 necrosis was found in 1 treated animal and in 4 controls. Steatosis was found in the grafts of 7 treated rabbits and in 8 controls. Tissue calcification was found in 8 treated animals and 7 controls. Therefore, since LDE-MTX markedly reduced the macrophage invasion (Table 2), the hallmark of the post-transplantation immune process, and prevented necrosis (25), it can be assumed that the LDE-MTX treatment improved the overall histological status of the grafts.

Again, the native hearts of both LDE-MTX and controls had apparently normal myocardium tissue, without inflammatory sites, and well defined and anatomically preserved arteries. Steatosis was not present in native hearts of either group.

### Gene expression

**Figure 2.**
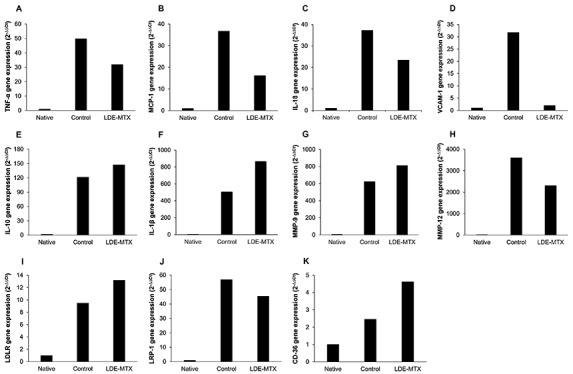
Effects of the treatment with lipid nanoemulsion-methotrexate (LDE-MTX) on gene expression of inflammation markers and lipoprotein receptors in grafted hearts. Gene expression of *TNF-α* (A), *MCP-1* (B), *IL-18* (C), *VCAM-1* (D), *IL-10* (E), *IL-1β* (F), *MMP-9* (G), *MMP-12* (H), *LDLR* (I), *LRP-1* (J), CD-36 (K), in control native and grafted hearts, and LDE-MTX grafted hearts of the rabbits. Total RNA was isolated from cardiac tissue and pooled samples of each group were reverse transcribed into cDNA. Quantitative real time PCR was used to calculate relative gene expression of target genes comparing the mean of the pool of grafted hearts of control and LDE-MTX groups versus the mean of the pool of native hearts of control group.

Gene expression levels in the grafted hearts of genes related with membrane receptors, inflammation, and metalloproteinase are shown in Figure 2.

Comparing the grafted hearts from the LDE-MTX with those of the control group, a remarkable effect of the treatment upon the gene expression of the pro-inflammatory factors was observed. LDE-MTX treatment elicited a twenty-fold reduction in both *TNF-α* and *MCP-1* gene expression and a fifteen-fold reduction upon *IL-18* (Figure 2A–C). The effect of the treatment upon *VCAM-1* gene expression was even greater, at the order of thirty-fold reduction (Figure 2D). Furthermore, LDE-MTX treatment elicited an increase in the gene expression of anti-inflammatory *IL-10* (Figure 2E). LDE-MTX, however, was ineffective in reducing the gene expression of pro-inflammatory *IL-1 β* (Figure 2F).

The gene expression of *MMP-9* and *MMP-12* of the heart grafts was strikingly higher than that measured in the native hearts. Comparing the grafted hearts from LDE-MTX treated with those from the controls, the treatment strongly decreased *MMP-12* while increasing *MMP-9* (Figure 2G–H).

Regarding the cell membrane receptors involved in the uptake of lipoproteins, the gene expression of the *LDLR* was roughly ten-fold higher in the heart grafts of both control and LDE-MTX treated groups of rabbits compared to the native hearts of the control animals. It was remarkable that the increase in gene expression of the *LRP-1* receptors in the grafted hearts, regardless if from the control or from the LDE-MTX treated group, was still much larger, at the range of forty to fifty-fold higher than that of the native hearts. There was also an increase in the gene expression of the *CD36* scavenger receptor in the grafted hearts that was, however, more discrete, at two- (controls) to four-fold (LDE-MTX treated) increase range (Figure 2I–K).

## Discussion

In this study in rabbits with heterotopic heart graft, LDE-MTX was effective in reducing the concentric stenosis of coronary arteries of the grafts, which is the hallmark of coronary allograft vasculopathy. It is worthwhile to point out that the native hearts were apparently not affected by the grafting of the heterotopic hearts in the various aspects examined here, such as inflammation, anatomic arterial status and steatosis. This finding excludes the possibility that cholesterol feeding was the sole factor to produce the above described alterations in the grafted hearts. The current experimental animal model thus approaches that of heart graft disease, wherein an immune-related process occurs in conjunction with atherosclerotic risk factors such as dyslipidemia (3).

The coronary artery stenosis that occurred in the heart grafts of the control animals predominantly consisted of cell proliferation in the intima layer, with some additional macrophage invasion. Thus, the marked stenosis reduction, of approximately 20%, with four-fold increase in vessel lumen area, achieved by LDE-MTX treatment was chiefly due to the anti-proliferative action of the drug. This action may have been effected by directly inhibiting cell mitosis or by suppressing macrophage secretion of cytokines. Such enlargement of the arterial lumen has the potential to strongly improve the myocardium perfusion and the graft dysfunction that ultimately leads to heart failure.

A key feature of organ rejection in heart transplantation is the intense macrophage invasion (25,26). The reduction of the macrophage staining in the heart grafts, observed in the LDE-MTX treated group compared with the controls, is thus indicative not only of the anti-inflammatory capabilities of this therapeutic approach but also of the eventual potential of LDE-MTX to prevent rejection. Necrosis, which is the most graft-damaging process, was also clearly attenuated by LDE-MTX treatment.

The gene expression pattern of pro and anti-inflammatory mediators, documented here after the LDE-MTX treatment by comparison with the controls, is highly suggestive that the novel preparation acts through inhibition of pro-inflammatory factors, such as *TNF-α, IL-18, VCAM-1* and *MCP-1* and stimulation of the gene expression of anti-inflammatory factors such as *IL-10*. However, the gene expression of pro-inflammatory *IL-1β* unexpectedly increased after the LDE-MTX treatment.

In previous studies, the toxicity of LDE-MTX compared to MTX was documented in mice (19). Here, in comparison with the control rabbits, LDE-MTX treatment did not result in observable toxicities that could be ascribed to the drug, since the rabbits did not show weight loss, differences in chow consumption or hematological changes such as anemia, neutropenia or lymphopenia.

Although the introduction of cyclosporine A was a turning point in the management of the post-transplantation condition, the drug primarily acts on the control of cell rejection but is ineffective in improving graft vasculopathy. On the other hand, the life-long immunosuppressive therapy to control graft rejection, which, as a standard, consists in cyclosporine A, azathioprine and corticosteroids, may bear side-effects such as hepatotoxicity and nephropathy (27). In this setting, there is a quest for new agents that could improve the control of rejection and graft vasculopathy (28–31). Drug delivery strategies, such as the LDE system used in this study, aim to improve the efficacy, selectivity and safety of therapeutic agents (32). The remarkable increase in MTX uptake by cells endowed by the association to LDE may have been accounted, at least in part, for the marked results in heart-grafted animals found in this study.

To the best of our knowledge, our previous study on LDE-paclitaxel used in heart-grafted rabbits was the first to use a drug delivery system to control the effects of organ transplantation (10). As LDE-paclitaxel remarkably ameliorated the grafting vessel damage with virtually no toxicity, similarly to the current LDE-MTX preparation, it is tempting to use the combined treatment with the two LDE preparations aiming to obtain additive or even synergistic effects. Simultaneous treatment with LDE-MTX and LDE-etoposide administered to rabbits with atherosclerosis induced by cholesterol feeding resulted in increased reduction of the lesions compared to the isolated use of the two preparations (33).

In conclusion, the data of this study indicate that the feature most characteristically associated to cardiac allograft vasculopathy, namely vessel stenosis, was strongly ameliorated by the LDE-MTX treatment, along with the grade of necrosis and markers of the inflammatory process. In view of the current lack of effective treatments for cardiac allograft vasculopathy, there is a compelling opportunity for the use of LDE-MTX in clinical trials as a promising therapeutic tool.
